# D-glucuronyl C5-epimerase acts in dorso-ventral axis formation in zebrafish

**DOI:** 10.1186/1471-213X-5-19

**Published:** 2005-09-12

**Authors:** Giancarlo Ghiselli, Steven A Farber

**Affiliations:** 1 Department of Pathology and Cell Biology, Thomas Jefferson University, 1020 Locust Street, Philadelphia, PA 19107, USA; 2 Kimmel Cancer Center, Thomas Jefferson University, 1020 Locust Street, Philadelphia, PA 19107, USA; 3 Department of Microbiology and Immunology, Thomas Jefferson University, 1020 Locust Street, Philadelphia, PA 19107, USA

## Abstract

**Background:**

Heparan sulfate (HS) is an ubiquitous component of the extracellular matrix that binds and modulates the activity of growth factors, cytokines and proteases. Animals with defective HS biosynthesis display major developmental abnormalities however the processes that are affected remain to be defined. D-glucuronyl-C5-epimerase (Glce) is a key HS chain modifying enzyme that catalyses the conversion of glucuronic acid into iduronic acid, a biosynthetic step that enhances HS biological activity. In this study the role of Glce during early zebrafish development has been investigated.

**Results:**

Two Glce-like proteins (Glce-A and -B) are expressed in zebrafish at all times. They are the products of two distinct genes that, based on chromosomal mapping, are both orthologues of the same single human gene. Transcripts for both proteins were detected in fertilized zebrafish embryos prior to the onset of zygotic transcription indicating their maternal origin. At later developmental stages the epimerases are expressed widely throughout gastrulation and then become restricted to the hindbrain at 24 h post-fertilization. By monitoring the expression of well characterized marker genes during gastrulation, we have found that misexpression of Glce causes a dose-dependent expansion of the ventral structures, whereas protein knockdown using targeted antisense morpholino oligonucleotides promotes axis dorsalization. The ventralizing activity of Bmp2b is enhanced by Glce overexpression whereas Glce knockdown impairs Bmp2b activity.

**Conclusion:**

Glce activity is an important determinant of of dorso-ventral axis formation and patterning in zebrafish. In particular Glce acts during gastrulation by affecting Bmp-mediated cell specification. The results obtained further corroborate the concept that HS encodes information that affect morphogenesis during early vertebrate development.

## Background

Heparan sulfate proteoglycans (HSPG) are macromolecules found in all connective tissues, extracellular matrices and on the surface of cells [1]. Their most prominent feature is the presence of one or more heparan sulfate (HS) chains covalently attached to a core protein [2]. The heterogeneity of the HSPG is due to the variation of the core protein, as well as to the type and size of the HS chains. Configuration variation in the disaccharide bonds and the position of sulfation leads to increased diversity in the chemical and structural properties of these chains. HS is composed of repeating disaccharide units of D-glucuronic acid (GlcA) or L-iduronic acid (IdoA) both of which may be 2O-sulfated, and unsubstituted, N-acetylated, or N-, 3O- or 6O-sulfated glucosamine (Glc). Forty-eight different disaccharides are possible but because of constraints in the biosynthetic process, only 23 have been identified in HS as biosynthetic intermediates [3]. Typical HS chains contain relatively short segments of modified sequences represented by IdoA-GlcNS derivatives of different sulfation content dispersed among large sections of unmodified (GlcA-GlcNAc) units.

The biosynthesis of HS occurs in the Golgi and involves the sequential modification of the nascent polysaccharide chain [4,5]. The conversion of GlcA into IdoA is a critical modification mediated in mammals by a single enzyme: D-glucuronyl-C5-epimerase (GLCE) [4,6]. Epimerization of GlcA increases the flexibility of HS chain thereby enhancing its ability to interact with proteins [2,7-9]. IdoA is the preferential substrate of the HS 2-O-sulfotransferase. Disaccharide units containing IdoA-2-O-S are organized in clusters along the HS chain and are specifically recognized by growth factors and morphogens [5,10]. The essential role played by Glce during development is demonstrated by the fact that transgenic mice that are *Glce*-null generally express highly abnormal HS structures and die neonatally [11,12]. *C. elegans *expressing mutated Glce, display abnormal neuronal development characterized by specific axonal and cellular guidance defects [13].

Much of the information concerning the role of HS in development has been obtained from studies in *D. melanogaster *[14]. An important concept arising from those studies is that the establishment of a morphogen gradient necessary for early patterning requires HSPG. This function is likely to involve the polysaccharide chain since morphogens such as Wingless (Wg) [15], Decapentaplegic (the orthologe of the vertebrate bone morphogenetic protein 4, Bmp4) [16,17], Hedgehog (Hh) [18] and several fibroblast growth factors (FGFs) [19] bind to HS. The specific role of HS in vertebrate development however remains conjectural and the developmental mechanisms that are affected have not been clearly identified. In zebrafish, lack of uridine 5'-diphosphate glucose dehydrogenase [20], an enzyme required for the biosynthesis of extracellular matrix glycosaminoglycans including HS, affects bone and heart morphogenesis. In mice [21] and zebrafish [22] the disruption of HS biosynthesis affects the nervous system development that can be ascribed to the effect HS has on the activity of multiple morphogens. In this paper we report that Glce's activity affects the establishment of the embryonic dorso-ventral (D/V) axis through a mechanism involving the bone morphogenetic proteins (Bmps).

## Results

### Cloning and chromosomal mapping of zebrafish *glce*

**Figure 1:**
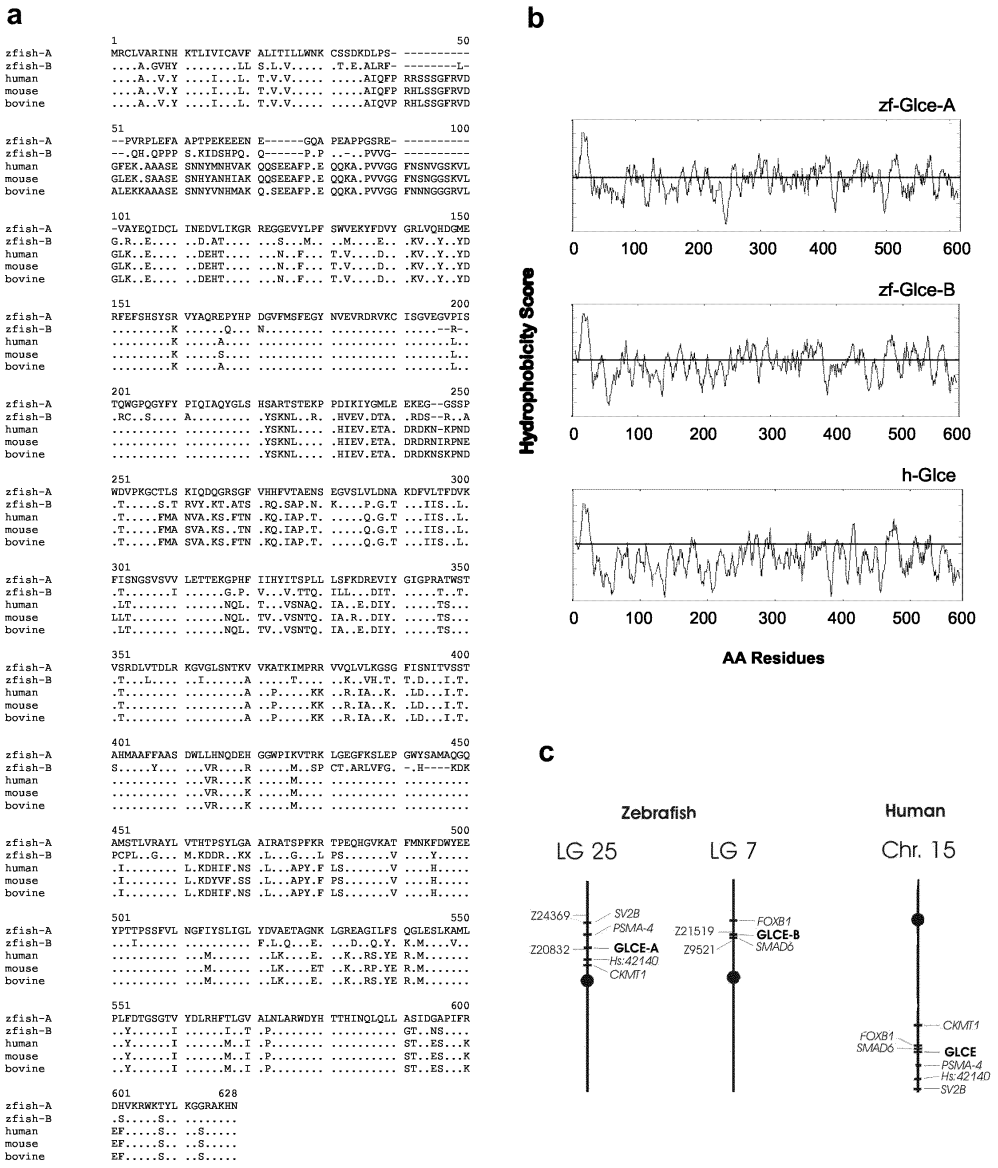
***Cloning and structural analysis of zebrafish glce***. (a) Alignment of Glce from zebrafish, human, mouse, and bovine. Conserved AAs are dotted. (b) Hydrophobicity plot of zebrafish and human Glce. Values above the line represent positive hydrophobicity scores. (c) Chromosomal mapping of zebrafish *glce-A *and *glce-B *and homology to the human *Glce *locus.

Zebrafish *glce*-A and *glce*-B genes both encode proteins of 585 amino acids. The gene products are homologous to the human protein sequence (67% and 73% respectively) (fig. 1a). Compared to the human, mouse and bovine sequences, the zebrafish proteins lack part of the N-terminus. The C-terminal domain is the most conserved region of Glce. Analysis of the hydrophobicity index determined utilizing the SOSUI [23] and the TMPRED [24] algorithms, reveals the presence in both zebrafish proteins of a conserved hydrophobic domain of ~20 amino acids located between residue 10 and 30 at the N-terminus of the proteins (fig. 1b). As the mammalian enzyme, zebrafish epimerase is a "type-two" transmembrane protein with predicted localization in the Golgi apparatus [4]. The *glce*-A and the *glce*-B locus mapped to linkage group LN25 between markers Z24369 and Z20832 and to linkage group 7 between markers Z21519 and Z9521, respectively (fig. 1c). Based on the mapping of neighbor genes, both chromosomal regions are synthenic with human chromosome 15q22, i.e. the region harboring the epimerase gene.

### Glce is maternally and zygotically derived

**Figure 2:**
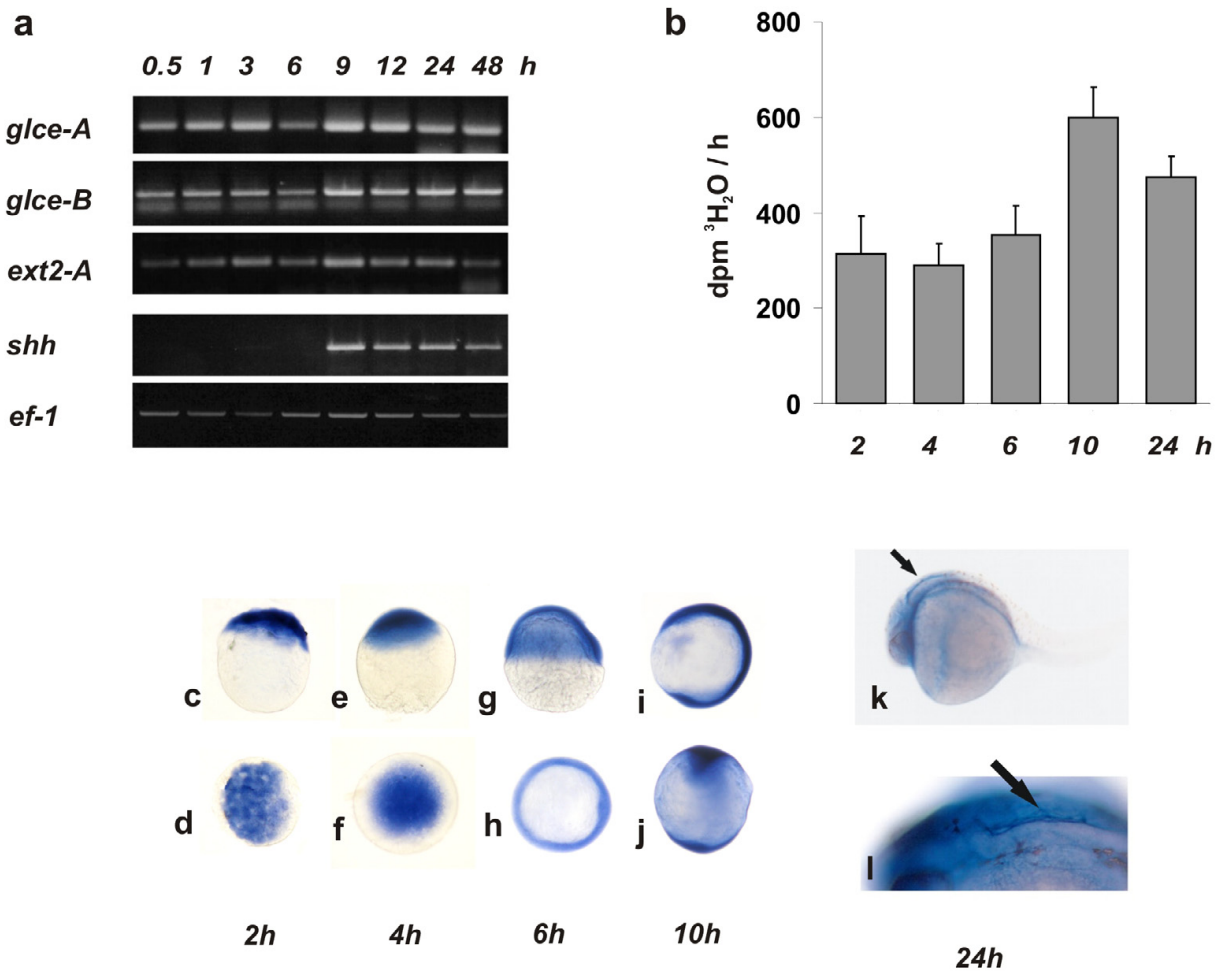
***glce mRNA expression pattern in developing embryos***. (a) RT-PCR analysis of the transcript level. Thirty embryos were collected in Tri-Reagent at different developmental stages as indicated. cDNA was generated from total RNA (1 μg) using Sensiscript reverse transcriptase primed with oligo-dT at 37°C for 2 h. PCR reactions (25 cycles) were performed in duplicate and analyzed on 1% agarose gel. (b) Glce enzymatic activity in embryos at different developmental stage. At each time point 20 embryos were dechorionated and homogenized. For the enzymatic assay, a cell lysate was incubated (2 h at 28°C) with labeled bacterial K5 heparosan substrate and the ^3^H_2_O liberated as result of the epimerization of GlcA into IdoA, measured. The bars represent the mean ± SD of the values from three independent determinations. (c-l) whole-mount *in-situ *hybridization of *glce-a *in embryos at different stages of development. (c-j) Top row: lateral views. Bottom row: animal pole views. (c,d) blastoderm at 64 cells stage; (e,f) dome stage; (g,h) shield stage; (i,j) 3 somite stage. (k) 24 hpf embryo showing showing intense *glce *staining at the perimeter of the forth ventricle as indicated by the arrow-heads. (l) enlargment of the embryo brain forth ventricle area.

*glce* transcript level was examined at different developmental stages and compared to that of *ext2-A*, an HS polymerase acting upstream the epimerization step [3]. The expression of s*hh *which is activated during gastrulation, and that of *ef-1 *which is expressed at similar level throughout development, were also monitored. *glce*-A, *glce*-B and *ext2-A* transcripts were present in fertilized embryos at developmental stages prior to the onset of zygotic transcription, indicating that these messages are maternally derived. The expression of HS biosynthesis enzymes reached a peak at the onset of gastrulation following midblastula transition (fig. 2a). Assay of epimerase activity in embryonic extracts at different developmental stages, was consistent with the level of mRNA encoding these proteins. In particular epimerase activity at 10 hpf was twice that observed at the 64-cells stage (fig. 2b).

### Temporal and spatial expression of Glce

*glce *transcripts were localized in embryos at different developmental stages by *in-situ *hybridization using gene-specific antisense riboprobes (fig. 2c–l). During the early stages a diffuse staining was observed throughout the blastoderm. At the beginning of segmentation, staining was detected along the entire dorsal axis (fig. 2i,j). At 24 hpf, however, g*lce *expression was higher in the newly forming brain (fig. 2k). At this site epimerase transcripts were mostly detected at the perimeter of the forth ventricle (fig. 2l). The staining specificitity was confirmed through the use of sense control riboprobes in which case only background staining was seen. No significant differences were detected in the expression pattern of the two g*lce *genes.

### Overexpression of Glce causes ventralization and potentiates Bmp activity

**Figure 3:**
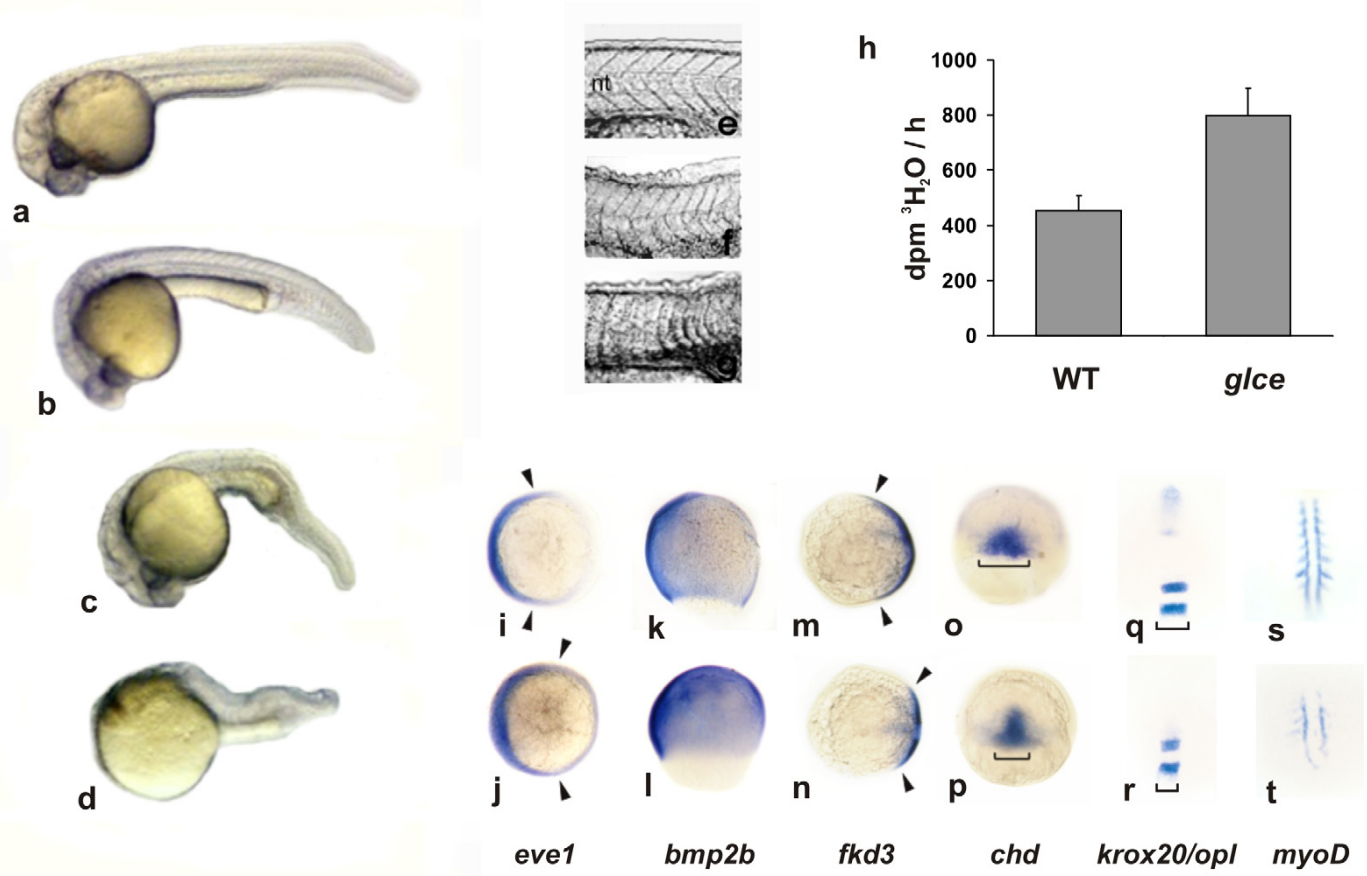
***Effect of Glce overexpression on embryo morphogenesis and HS composition***. Embryos at 1–2 cells stage were injected with *glce-A *and/or *glce-B *mRNA and observed at 24 hpf. (a) wild type embryo. (b,c) mild and moderately ventralized embryos showing enlarged blood sac (indicated by an arrow). (d) Severely ventralized embryos displaying dramatically reduced head and trunk. (e-g) High-contrast images of the somites and the notocord (nt) structures in control (e), and in mildly and moderately ventralized embryos (f,g). Note the loss of chevron-like structure of the somites and the narrowing or disappearance of the notocord in embryos overexpressing *glce *(f,g). (h) Epimerase enzymatic activity at 10 hpf in controls (WT) and in embryos injected with *glce-A *plus *glce-B *mRNA (250 pg each). The enzymatic assay was performed as described in Fig. 2b. (i-t) Whole mount *in-situ *hybridization with D/V markers in embryos during gastrulation and at 5 somite stage. Top row: wild type embryos. Bottom row: embryos injected with 250 pg each of *glce-A *and *glce-B *mRNA. (i,j) *eve1 *staining viewed from the animal pole at shield stage. The arrowheads point to the edges of the expression range of the marker; (k,l) *bmp2b*, lateral view with the dorsal side to the right at 70% epiboly; (m,n) *fkd3*, animal pole view at 70% epiboly; (o,p) *chordino*, dorsal view at 50% epiboly; (q,r) *krox-20*/*opl *double staining (head view) and (s,t) *myo-D *(dorsal view) in embryos at 5 somite stage. Note in (r) the narrow expression domain of *krox20 *in embryos injected with *glce *mRNA whereas *opl *transcript is undetectable.

To investigate the functional significance of the epimerase during zebrafish development the protein was ectopically expressed by injecting embryos (1–2 cell stage) with capped *in vitro*-transcribed mRNA. The majority of injected embryos displayed a ventralizing phenotype whose severity correlated with the dose of mRNA injected (250 to 1000 pg) (table 1). The affected embryos had smaller head size, expanded blood islands, and abnormal tail somites (fig. 3b,c,f). More strongly ventralized embryos also lacked a notochord and developed somites that were not chevron-shaped and were fused in the midline below the neural tube (fig. 3g). Overexpression of *glce-B *produced an identical spectrum of phenotypes as the overexpression of *glce-A*. However, the highest frequency of severely affected embryos was observed when 250 or 500 pg of *glce-A *and *glce-B *mRNA were administered together in which case most of the animals failed to form an anterior axis (fig. 3d). In this treatment group, epimerase enzymatic activity at 10 hpf was 73% higher the level detected in uninjected embryos (fig 3h).

**Table 1:** Effect of the ectopic expression of Glce on Bmp2b ventralizing activity. Capped *glce *and *bmp2b *mRNAs were generated by reverse transcription from full length cDNA clones. After extraction in phenol/chloroform and precipitation in isopropanol, mRNA was dissolved in Danieau's buffer and the concentration assessed by UV reading (260 nm). Embryos at one or two cell stage were injected with 1–3 nl of mRNAs to achieve the indicated dose. Each injection session consisted of 2–3 treatment groups of 30 embryos each and several experiments were performed to reach the sample number indicated. Embryos with increasing degree of ventralization were ranked according to previously established criterias [40,41]. Ventralized V1 embryos show reduced eye size and shorter body. In addition to these abnormalities V2 embryos display abnormal notocord, reduced anterior somites, and enlarged blood islands. Ventralized V3 embryos have little or no head structures and no notochord. V4 embryos display gross body abnormalities and lack of anterior structures.

**Injected mRNA**	**Strength of Ventralization**					
	**No.**	**Wild Type**	**V1**	**V2**	**V3**	**V4**

		(%)	(%)	(%)	(%)	(%)

Uninjected	120	100	0	0	0	0
*glce-A *(250 pg)	60	65	23	10	2	0
*glce-B *(250 pg)	60	54	16	28	2	0
*glce-A *(125 pg) *glce-B *(125 pg)	30	60	7	23	10	0
*glce-A *(250 pg) *glce-B *(250 pg)	30	37	6	10	47	0
*glce-A *(500 pg) *glce-B *(500 pg)	60	26	0	38	35	0
*bmp2b *(20 pg)	50	0	26	37	26	11
*bmp2b *(20 pg) *glce-A *(250 pg)	60	0	0	15	45	40
*bmp2b *(20 pg) *glce-B *(250 pg)	30	0	0	26	44	30

In order to better characterize the phenotype of embryos overexpressing Glce, the expression of dorsal and ventral markers were analyzed by *in situ *hybridization [25,26]. The expression domain of *eve1*, a marker of ventral and lateral regions at early gastrula stages, was expanded at the shield stage (fig. 3i,j). Likewise the range of expression of *bmp2b *was greatly enlarged in embryos at the 70% epiboly stage (fig. 3k,l). In contrast expression of *fkd3*, a marker of the presumptive neuroectoderm, was reduced by Glce overexpression (fig. 3m,n). Similarly the expression domain of *chordin*, a marker of the dorsal organizer, was reduced (fig. 3o,p). The fact that overexpression of the epimerase alters the pattern but does not prevent the expression of dorsal determinants, is consistent with the idea that Glce acts on D/V axis formation downstream the Wnt/β-catenin pathway that regulates *chordin *gene expression [27]. *glce *is also a target of the Wnt/β-catenin transactivation pathway [28] raising the possibility that zygotic *glce *expression is coordinated with that of other D/V determinants.

Because head size is affected following ectopic expression of *glce *(fig 3b–d) the expression pattern of the neuroectoderm-specific markers *krox 20 *[29] and *opl (zic1) *[30] was determined during somitogenesis. The expression of *myoD*, a transcript specifically localized to somitic mesoderm was also examined [31]. During somitogenesis, *krox-20 *is normally expressed in hindbrain rhombomeres R3 and R5 both of which are dorsal ecdoderm derivatives. A reduced area of *krox-20 *expression was detected at the 5 somite stage in most of the embryos injected with 250 pg each of *glce-A *and *glce-B *mRNA whereas *opl *expression was undetectable (fig. 3q,r). *myoD *expression in the cells adjacent to the axial mesoderm and in the somitic furrows was also reduced implying a role of Glce in establishing mesodermal cell fate (fig 3s,t).

Since both the phenotype and marker gene expression pattern following ectopic expression of Glce is reminiscent of that of *chordino *[30,32-34], *ogon *[33,35,36] and *radar *[37,38] mutants or of embryos misexpressing *alk 8 *[39] in which the ventralizing activity of Bmps is enhanced, we examined whether Bmp2b activity is potentiated by Glce. For this purpose we titrated the dose of injected *bmp2b *mRNA to achieve a preponderance of partially ventralized embryos displaying V2 and V3 phenotype severity [40,41] (table 1). This same dose (20 pg) was then administered together with *glce-A *and/or *glce-B *mRNA (250 pg). Following the treatment, the majority of embryos exhibited the most severe V4 class phenotype consistent with Glce activity potentiating the effect of Bmp2b.

### Glce availability affects Bmp-mediated ventralization

**Figure 4:**
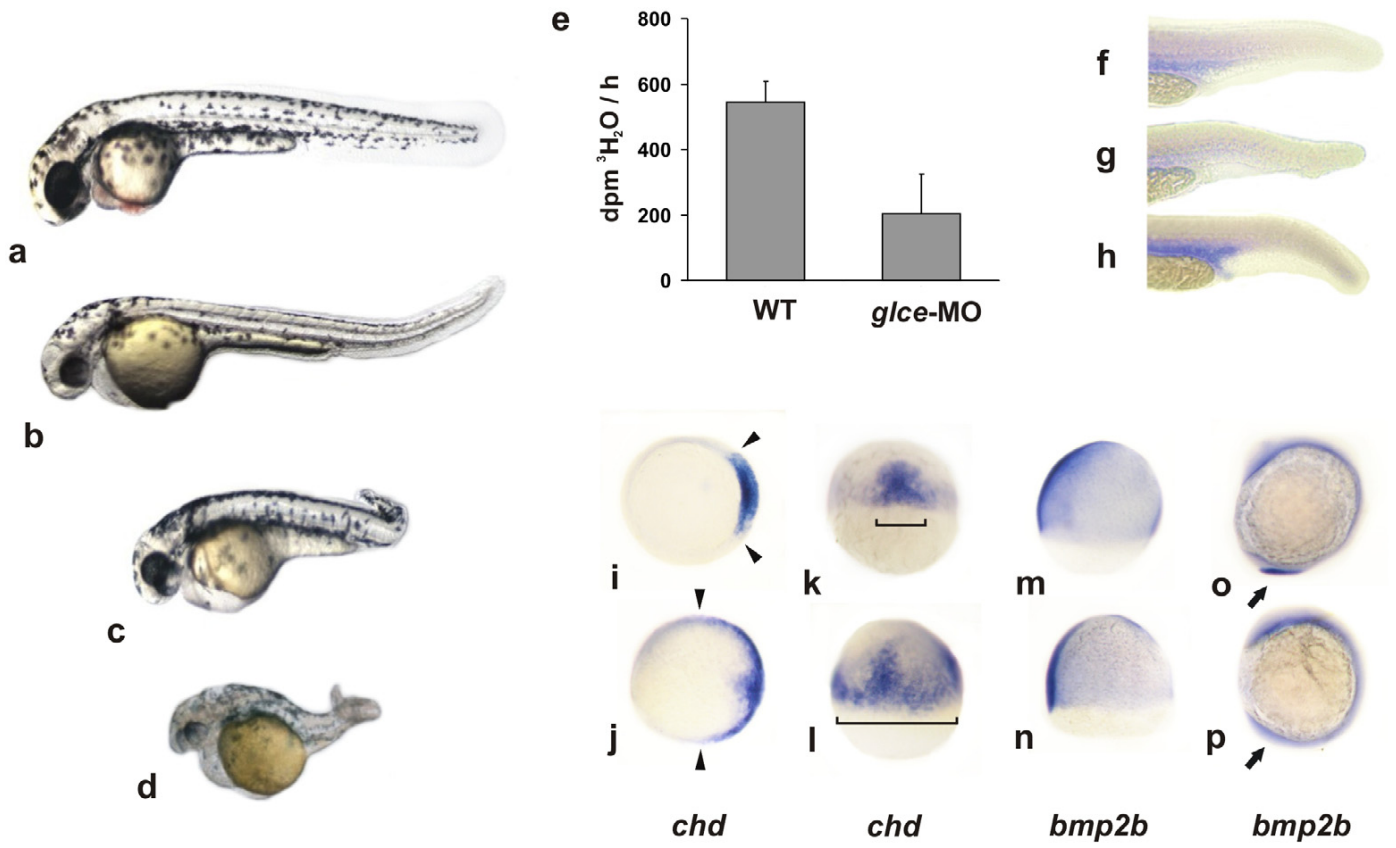
***Effect of Glce knockdown on embryo morphogenesis***. (a-d) Embryos at 1–2 cells stage were injected with a mix (8 ng each) of *glce-A *and *glceB *MO and the phenotype scored at 48 hpf. (a) wild type embryo. (b,c) mild phenotypes displaying reduced head volume and ventral fin extension. (d) severe phenotype with shortened A/P axis and loss of ventral structures. (e) Epimerase enzymatic activity in 10 hpf embryos and effect of Glce knockdown. The enzymatic assay was performed as described in Fig. 2b. (f-p) Whole mount *in-situ *hybridization with D/V markers of embryos at different developmental stages. (f) *gata1 *expression in wild-type embryos, (g) *glce *morphants, and (h) in embryos overexpressing *glce *at 24 hpf. (i-l) *chordino *at 50% epiboly in wild type (top) and morphants (bottom). (i,j) animal pole view, (k,l) dorsal view. (m,n) b*mp2b *at 70% epiboly and (o,p) at 3 somite stage in wild type (top) and morphants (bottom). Note the absence of b*mp2b *expression in the presumptive tail of the morphants as indicated by the arrows.

The effect of Glce protein knockdown on D/V axis formation was next examined by administering antisense morpholino oligonucleotides (MO) targeting *glce-A *and *glce-B *transcripts. Most of the embryos after injection of 4 ng of antisense MO, displayed a mild dorsalized phenotype with reduced ventral tail fin (fig. 4b and table 2). At higher dose (8 ng) about half of the morphants showed kinked tail, enlarged heart cavity and in some animals the atrioventricular boundaries failed to form (fig. 4c). A dramatic shortening and reduction of the body mass with tail coiling similar to the phenotype associated with mutation of Bmp2b, and Bmp7 [34,41] was observed in the majority of the embryos receiving the highest dose (16 ng) of MO (fig. 4d). In this group of morphants the epimerase enzymatic activity was significantly decreased (34% of the control at 8 hpf) (fig. 4e). The inability of the MOs to completely abolish the Glce activity suggests that at this time residual maternally derived enzyme is still active. In spite of this, the effect of Glce knockdown on ventral cell fate was already detectable in the mild morphants as revealed by the faint staining of *gata1 *expressing blood islets (fig. 4g), a ventral tissue derivative [42]. In stronger morphants, a broadening of the *chordin *expression domain wasobserved (fig. 4i–l). In addition, consistent with the axis dorsalization, the expression of *bmp2b *at shield stage was severely reduced as also evidenced by its undetectable expression in the tail during somitogenesis (fig. 4m–p). The administration of human or zebrafish *glce *mRNAs to embryos injected with MO rescued the enzymatic activity and prevented the development of the most severe dorsalized phenotypes (table 2).

**Table 2:** Effect on D/V axis formation of antisense targeting of *glce*. Capped mRNA and *glce*-MOs were dissolved in Danieau's buffer and injected as 1–3 nl bolus into the yolk of one- to two-cell embryos as described in Table 1. Capped human *GLCE*-mRNA was generated from a full length cDNA clone. The embryos were classified according to the severity of the observed phenotype at 48 hpf based on a classification of the dorsalization severity observed in embryos that had only received MOs. Mildly dorsalized embryos show reduced ventral tail fin extension. Moderately dorsalized embryos display kinked tail, enlarged heart cavity and in some case the absence of atrioventricular boundary. Severely affected morphants show dramatic shortening and reduction of the body mass with tail coiling. For the rescue experiments, embryos were first injected with MO and after randomization half received the indicated amount of mRNA before reaching the 4-cell stage.

**Treatment**	**Phenotype Severity**				
	**No.**	**Wild**	**Mild**	**Moderate**	**Severe**

		(%)	(%)	(%)	(%)

Uninjected	42	100	0	0	0
*glce-A *MO (4 ng)	62	35	46	19	0
*glce-A *MO (8 ng)	40	20	25	39	16
*glce-A *MO (16 ng)	28	12	19	31	38
*glce-B *MO (16 ng)	30	25	19	30	26
*glce-A *MO (8 ng) *glce-B *MO (8 ng)	30	15	10	30	45
*glce-B *MO (16 ng) *glce-A *mRNA (200 pg)	68	28	38	22	12
*glce-B *MO (16 ng) *glce-B *mRNA (200 pg)	30	32	45	15	8
*glce-A *MO (16 ng) Human *GLCE *mRNA (200 pg)	35	21	41	29	9

In order to assess the dependency of Bmp activity on Glce level, embryos were injected with either 50 pg of *bmp2b *mRNA or 100 pg of *bmp4 *to generate a preponderance of V3-V4 ventralized embryos (table 3). Following randomization, half of the injected embryos received a mix of MOs targeting both *glce-A *and *glce-B *transcripts. About two-third of the embryos receiving the MOs displayed a normal-to-mild (V1-V2) ventralized phenotype whereas few developed the most severe V4 class phenotype. These results are in stark contrast to the embryos that had only received *bmp2b *and *bmp4 *mRNA supporting the concept that Glce is required for Bmp-mediated ventralization.

**Table 3:** Effect of antisense MO on the ventralizing activity of Bmps. Capped mRNA and *glce *MOs were injected into the yolk of one- to two-cell embryos as in the experiments of table 1. The resulting phenotypes were scored at 48 hpf. For the rescue experiments, embryos were first injected with *bmp2b *or *bmp4 *mRNA and after randomization half received the indicated amount of MO before reaching the 4-cell stage. Phenotype severity was scored as in Table 1.

**Treatments**	**Strength of Ventralization**					
	**No.**	**Wild**	**V1**	**V2**	**V3**	**V4**

		(%)	(%)	(%)	(%)	(%)

Uninjected	40	100	0	0	0	0
*bmp2b *mRNA (50 pg)	62	0	0	6	18	76
*bmp2b *mRNA (50 pg) *glce-A *MO (8 ng) *glce-B *MO (8 ng)	60	15	15	31	25	16
*bmp4 *mRNA (100 pg)	60	0	0	0	15	85
*bmp4 *mRNA (100 pg) *glce-A *MO (8 ng) *glce-B *MO (8 ng)	60	21	18	23	23	15

## Discussion

In mammals, HS plays a crucial role in a variety of important biological processes including the regulation of blood coagulation, cell adhesion and differentiation, angiogenesis, and virus infection [1,3,43]. Most of the information concerning the role of HSPG in development has been obtained in the invertebrate model organism *D. melanogaster *and support the idea of a major functional role for HS in the morphogen's gradient establishment [3,14,44,45]. The fly mutants *Sugarless *[46], *fringe connection *[47], *sulfateless *[48], *and tout-velu *[49,50], display cuticle abnormalities that are reminiscent of the phenotypes exhibited by the mutations in Wg, Hh, or FGF and suggest an involvement of HS in the activity of these morphogens. In *Drosophila *the lack of HS also affects the body axis formation, but this effect is evident only at later stages of development [14]. Compared to the wealth of data generated in invertebrate species, the functional role of HSPG in vertebrate development is still poorly investigated. Transgenic mice carrying null-mutations in genes coding for enzymes implicated in HS chain polymerization [51], glucosamine N- or IdoA 2O-sulfation [52,53], and GlcA epimerization [11] are not viable leading to the conclusion that HS encoding critical structural epitopes is required for normal embryonic development [54]. The developmental mechanisms affected by the lack or by structurally aberrant HS, however remain to be assessed.

In this study the specific role of Glce has been investigated. GlcA epimerization endows the nascent polysaccharide HS with increased biological activity and is necessary to direct further chain sulfation at specific sites [3,6]. In mammals the enzyme is a single gene product whereas in zebrafish two genes have been identified likely arising from gene duplication. Transcripts for the two epimerases are already detected during the early cell divisions indicating a maternal contribution to the zygotic pool. The temporal expression pattern of g*lce *closely resemble that of e*xt2-A *suggesting that HS chain elongation and GlcA epimerization may be activated at the same time. Up to 12 hpf *glce* expression is detected along the entire axis. At 24 hpf however, the epimerase is highly expressed in the hindbrain, most notably along the perimeter of the fourth ventricle. In the hindbrain, at this developmental stage, Glce may play a specialized role involving axonal guidance as postulated based on observations made in *C. elegans *with mutated *glce *[13]. It will be of interest in future studies to compare the pattern of expression of *glce *and *ext2 *with that of the other enzymes involved in polysaccharide chain formation and sulfation to test the hypothesis that HS structure is developmentally regulated. For example zebrafish glucosamine 6O-sulfotransferase which act downstream to the biosynthetic step catalyzed by Glce, is not maternally derived [55] suggesting that GlcA epimerization and glucosamine sulfation represents two distinct pathways regulating HS structure during development.

The fact that the expression of Glce is rather ubiquitous throughout gastulation, has given us the opportunity to investigate the role of this enzyme by globally perturbing its level either by injecting capped mRNA or antisense oligonucleotides. When misexpressed the protein produced a ventralized phenotype similar to that observed in null-mutants for genes *ogon *[33,35,36], *radar *[37,38] and *chordino *[25,36] that directly modulate the function of the ventralizing agents Bmps albeit through different mechanisms. This phenotypic similarity lead us to focus on the role of Glce with respect to the activity of Bmps. During development the cell fate in zebrafish depends on the position within the embryo during blastula and gastrula stages. Positional information to cells are provided through the establishment of an activity gradient of Bmp proteins that promotes ventral specification in a dose dependent manner [40,56,57]. The idea that the specification of cells fate along the D/V axis is mediated through Bmp acting as terminal effectors, is supported by the fact that activation of the Bmp signaling pathway is a rather late event during embryogenesis and by the observation that functional inactivation of the zebrafish genes Bmp2b (*swirl*) [40] and Bmp7 (*snailhouse*) [34] both result in dramatic suppression of ventral fates and expansion of dorsal structures. Bmp2b and Bmp4 interact with HS through a cluster of positively charged aminoacids located at the N-terminus outside the receptor-binding domain of the protein [17]. Additional studies indicate that the interaction of Bmps with HS has important functional significance in that mutations in the HS/heparin binding domain results in an increase in the long-range activity of the morphogens [58]. HS also potentiates the biological activity of Bmps by serving the ligand to their receptor and/or by stabilizing the biological activity of the morphogen by preventing its proteolytic degradation [59]. Changes in IdoA content affecting HS binding to Bmp can thus change Bmp activity through different mechanisms. A spectrum of D/V mutants ranging from strongly ventralized to dorsalized embryos are generated when Glce activity is modulated suggesting that correct axial patterning requires that the activity of the epimerase be maintained within a critical range. An analysis of the structural domain in HS responsible for binding to Bmps can further elucidate what specific role IdoA residues play in this context.

Because HS ability to interact with proteins generally correlates positively with the IdoA content [2], Glce may be involved in the regulation of the activity of other heparin-binding morphogens involved in D/V axis formation. Fgf-8 has been demonstrated to play a key role is the establishment of D/V axis by acting upstream of Bmp2 and Bmp4 [60] and interacts with IdoA-rich regions in HS [61]. In zebrafish Fgf-8 inhibits the expression of Bmps in the ventral part of the embryo leading to the expansion of dorso-lateral derivatives at the expenses of ventral and posterior domains [60,62]. Based on our results an activation of Fgf-8 mediated pathway following ectopic Glce expression seems unlikely since an expansion rather than a reduction of ventral structures has been observed in embryos overexpressing the enzyme. It is possible that Glce overexpression inhibits Fgf-8 mediated signaling. This would occur if Fgf-8 is sequestered in the extracellular matrix by HS enriched in FGF binding regions or if the FGF receptor dimerization is negatively affected by HS [19]. However Fgf-8 null mutants display rather mild D/V abnormalities and similarly to *fgf-8 *morphants or mutant embryos with disrupted Ras/MAPK-mediated FGF signaling, do not form a midbrain-hindbrain boundary and do not develop the cerebellum [62]. Both these brain structures are present in the *glce *morphants and in embryos overexpressing *glce *unless, as a consequence of marked dorsalization or ventralization, the entire body plan is grossly altered. This finding is consistent with the observation that *Glce*-null mice have normal brain morphology [11] as it would be expected if Fgf-8 function is not affected [63]. Conceivably Fgf-8 mediated D/V patterning is little influenced by perturbation in Glce activity pointing to a downstream mediator of axis formation as sensitive to changes in HS IdoA content. A similar conclusion can be reached with regard to the D/V patterning effect of Wnt which acts upstream to Fgf-8, since activation of this pathway would result in posteriorization of the neural ectoderm affecting the eyes and the midbrain-hindbrain boundary development [64,65].

Taken together our results identify the stage of D/V patterning controlled by Bmp as sensitive to changes in HS structure. Previously it has been shown that mutants of the HSPG *Dally *have altered Dpp gradient formation resulting in abnormal patterning of the wing imaginal disc [16]. It was hypothesized that the HS chains of Dally bind Dpp and promote the interaction of the morphogen with the cognate cell surface receptor. Decreased interaction with HS, as may occurs when Glce activity is lowered, may reduce the concentration of Bmp available for interaction with the cognate receptors or the receptor-ligand binding is affected. Conversely, as a result of enhanced GlcA epimerization, HS affinity for Bmp may increase enhancing the concentration of the ligand at the receptor site and prolonging the morphogen activity [58,59,66]. The fact that Bmp is antagonized by proteins such as Chordin, Noggin, and Follistatin that require HS for diffusion and activation [67-69], represents an additional potential mechanism of regulation of Bmp activity that is dependent on HS. Chordin is required to dorsalize surrounding neuroectoderm and mesoderm and its expression pattern is affected when Glce activity is altered. A specific class of HSPG strongly potentiates the antagonism of Bmp signaling by Chordin and is necessary for the retention of Chordin by cells [69]. Likewise the interaction of Noggin with HSPG *in vivo *regulate its diffusion [67]. Conceivably in tissues rich of HS that binds with high affinity to Chordin and Noggin, the range of action of these Bmp antagonists is reduced and the ventralizing effect of Bmps may prevail. Our results support the hypothesis that correct D/V patterning depends upon the regulated expression of specific structural elements in HS and provide the basis for the interpretation of the functional role of Glce *in vivo*.

## Conclusion

The results obtained corroborate the concept that HS encodes information that directs morphogenesis during early vertebrate development. In particular Glce emerges from this study as an important modulator of vertebrate morphogenesis that acts in a dose-dependent fashion on D/V axis formation. Bmp-dependent cell fate specification is the main target of Glce activity. Glce effect may be mediated by potentiating the effect of Bmps or by restricting the range of action of other HS-binding proteins such as Chordin and Noggin that by antagonizing Bmps act as indispensable dorsalizing agents.

## Methods

### Zebrafish breeding and phenotype scoring

Embryos were obtained from natural mating of wild-type (Oregon, AB) fish and breed, raised and staged according to established criterias [70].

### Cloning of zebrafish Glce cDNA

A query of the zebrafish dEST database identified a number of putative clones whose translated sequence matched the N- and C-terminus of the human protein. Analysis of the predicted protein sequence of these clones indicated that zebrafish have two highly homologous Glce proteins. Cloning of the putative *glce *cDNAs was performed by reverse transcription of adult male zebrafish mRNA (Qiagen Sensiscript) primed with oligo-dT. cDNAs were amplified by PCR by combining primers matching the different possible cDNA terminal sequences. Forward primers were 5'-ATGCGCTGTCTGGTGGCTCGAATCAATC ACAAGACT-3' and 5'-ATGCGTTGTCTGGCAGCCGGTGTTCACTACAAGACC-3'. Reverse primers were 5'-CTAGTTGTGCTTAGCCCGACCTCCTTTCAGGTAAGT-3' and 5'-TTAATTGTGCTTAGCCCTCCCTCCTTTCAGGTAGCT-3'. This strategy resulted in two products of the expected size (~1.8 kb) from two of the four possible primer combinations. The amplified products were named *glce-A *(GenBank AY388516) and *glce-B *(AY388517), cloned into pcDNA3.1-TOPO (Invitrogen) and sequenced.

The 5'-end translated region of the zebrafish homologue of human exostosin 2 (*EXT2*) gene, was cloned using a similar strategy. The existence of two zebrafish *ext2 *genes were predicted from alignments of published EST sequences. The 5'-end of the *ext2-A *coding sequence including part of the UT region was cloned by RT-PCR using forward and reverse primers of sequence 5'-CATTCAACTTAAATATTCACCATA-3' and 5'-GGCGCTCAGCAGGTCATTGTATTC-3' respectively. Sequencing of an expected 528 bp fragment confirmed the identity of the amplified cDNA.

### Chromosomal mapping of zebrafish *glce*

In the human, the *glce *translational start site is located in a 602 bp exon. Assuming that zebrafish g*lce *genes maintain the same genomic organization as the human, PCR primers were designed to amplify the exon containing the translation initiation site for each each zebrafish orthologue. PCR amplification with primers 5'-ATGCGCTGTCTGGTGGCTCGAATC-3' and 5'-AGATGAAGGGCAGATACACCTCGC-3' for *glce-A *and 5'-ATGCGTTGTCTGGCAGCCGGTGTTCACTACAAG-3' and 5'-GACCTTTAATGGTGGCATCGTCATTGATCAGGC-3' for *glce-B *using genomic male zebrafish DNA as template, gave products of the expected size (420 bp and 261 bp for *glce-A *and *glce-B *respectively) that were cloned in pGEM-T vector and sequenced to confirm their identity. These sets of primers were then used to determine the chromosomal location of each gene by radiation hybrid panel (LN54) mapping.

### Antisense targeting of the transcripts

Antisense Morpholino oligonucleotides (MOs) (Gene Tools LLC) were designed to target the 5'-UT region of the genes of interest. *glce-A *and *glce-B *MOs had sequence 5'-AGCCATGAGGAACACGTCAGCAAAC-3' and 5'-TCCCTGCTTACCTGCAATGCAAACA-3', respectively. MOs were dissolved in water at a concentration of 4 mM and diluted in Danieu's buffer before injection.

### Generation of capped mRNA

Full-length *glce *cDNAs were subcloned in pT3TS vector at the *Bgl*II and *Eco*RV sites and *in vitro*-transcribed capped mRNAs were synthesized (T3 mMESSAGE mMACHINE kit, Ambion). mRNAs (1 μg) were tested prior to injection for protein expression *in vitro *using a rabbit reticulocyte lysate assay kit and ^35^S-methionine. Labeled proteins were separated on 9% SDS-PAGE gel followed by autoradiography. Human *glce *mRNA was generated from a full length cDNA clone in pcDNA 3.1 vector using T7 RNA polymerase. The human clone (AY635582) was obtained by RT-PCR using primers of sequence 5'-CTGCATATGCTGTGCTTGGCA-3' and 5'-CTAGTTGTGCTTTGCCCTGCTGCCTTT-3' based on published coding sequences and on cDNA 5'-end extension experiments we have performed [28]. *bmp2b *and *bmp4 *capped mRNA were kindly provided by Dr M. Mullins (U.Penn).

### Microinjection

MOs and mRNAs were injected (1–3 nl) into the yolk of 1–2 cell embryos [71]. Post-injection (6 h) embryos were sorted, the unfertile/damaged removed and the rest allowed to grow at 28°C for further observation.

### RNA *in situ *hybridization of zebrafish embryos

Antisense digoxigenin-labeled riboprobes were generated using SP6 or T7 RNA polymerase-based labeling kit (Roche). cDNA clones for *glce-a*, *glce-b*, *chordino*, *krox-20*, *opl*, *myoD*, were generated by RT-PCR [see Additional file 1]. *bmp2b*, *fkd3*, and e*ve1 *antisense riboprobes were generated by reverse transcription from cDNA clones (cb670, cb114, and cb872 respectively) obtained from the Zebrafish International Resource Center (University of Oregon). Whole embryo *in situ *hybridization was performed as previously described [72].

### Semi-quantitative RT-PCR

RNA was extracted and cDNA generated by reverse transcriptase using oligo-dT primers. An aliquot of the reaction was used as template for PCR amplification using gene-specific primers (Appendix I). Reactions were performed in duplicate and the product generated after 20 and 30 cycles analyzed by agarose electrophoresis to ensure that the products were quantitated during the exponential phase of the chain reaction.

### Epimerase enzymatic activity assay

Embryos were collected at the indicated times, dechorionated and washed in ice-cold 25 mM HEPES, pH 7.0 buffer containing 0.1% Triton X-100. Embryos were then homogenized in 200 μl of the same buffer with a pestle that fits tightly into an Eppendorf tube and stored at -70°C. The substrate for the epimerase enzymatic assay consisted of radiolabeled modified bacterial N-acetylheparosan prepared as described previously [28]. The final N-sulfated heparosan product was purified by ion-exchange chromatography and eluted at higher ionic strength than the starting bacterial polysaccharide (0.66 M vs. 0.40 M NaCl). The epimerase enzymatic assay was performed as described by Crawford et al. [4]. Briefly, reactions were set up by combining the homogenates from 20 embryos with labeled substrate (1 nmole ~30,000 dpm) followed by 2 h incubation at 28°C. Reactions were stopped by addition of DEAE-Sepharose (1 ml) equilibrated in 50 mM Na-acetate, 50 mM NaCl pH 4.0 buffer (1:1 volume) followed by 15 min incubation at 4°C. Tritiated water generated as a result of the epimerization of GlcA into IdoA was recovered in the supernatant following centrifugation at 10,000 rpm. The sample and a 800-μl rinse of the DEAE slurry with buffer, were counted for the associated radioactivity in a Wallac 1219 Rackbeta liquid scintillation counter. Values were corrected for a reaction blank obtained by adding the substrate to the embryo homogenate just prior to the addition of DEAE-Sepharose.

## Authors' contributions

GG cloned the zebrafish *glce *gene and the cDNAs used in the study, generated the capped mRNAs, performed the Glce enzymatic assay, and drafted the manuscript. SAF supervised the zebrafish injection experiments, provided training regarding the whole-mount *in situ *hybridization and performed some of the microscope analysis.

## Supplementary Material

Additional file 1 **Oligonucleotide primers **Primers used for semiquantitative RT-PCR analysis and for the generation of riboprobes Click here for file
