# Supplementary material for: D-glucuronyl C5-epimerase acts in dorso-ventral axis formation in zebrafish
Source: BMC Dev Biol. 2005 Sep 12;5:19. doi: 10.1186/1471-213X-5-19 (PMC1250224; doi:10.1186/1471-213X-5-19)
Supplement: Additional file 1 — Oligonucleotide primers Primers used for semiquantitative RT-PCR analysis and for the generation of riboprobes [file 1471-213X-5-19-S1.doc]

**APPENDIX 1**

**Oligonucleotide primers**

*glce-A* ATG CGC TGT CTG GTG GCT CGA ATC

AGA TGA AGG GCA GAT ACA CCT CGC

*glce-B* ATG CGT TGT CTG GCA GCC GGT GTT CAC TAC AAG

GAC CTT TAA TGG TGG CAT CGT CAT TGA TCA GGC

*ext2-A* CAT TCA ACT TAA ATA TTC ACC ATA

GGC GCT CAG CAG GTC ATT GTA TTC

*ef1* TTG AAA CTT ATC AAT CAT GGG AAA GG

AAA TTC ACT TGG TCT TGG CAG CC

*shh* AGA TTC AGC GTG CGG CAA AAT GCG

CCA CAG GAG GGT TTT CAG CTT GA

*chd* GTT GAA GGT GGT GTG GAT GAT GGA

TTA GAG AGT GTG AGT GTC TCC AGC

*myo-D* AAC ACA AAA AAC ATG GAG TTG TCG GA

GTT GCA GAA TTT TAA AGC ACT TGA TA

*opl* AAA TAG ATG CTC TTG GAC GCA GGA

TGA AAA TAT TTA CAC GTA CCA TTC

*krox-20* GAC TTC ACG ATG ACA GAT AAA ACT

TTG AAA GTT CAC TGG TTT GAA CTG
